# Reconstruction of ARNT PAS-B Unfolding Pathways by Steered Molecular Dynamics and Artificial Neural Networks

**DOI:** 10.1021/acs.jctc.0c01308

**Published:** 2021-03-29

**Authors:** Stefano Motta, Alessandro Pandini, Arianna Fornili, Laura Bonati

**Affiliations:** † Department of Earth and Environmental Sciences, University of Milano-Bicocca, Milan 20126, Italy; ‡ Department of Computer Science, Brunel University London, Uxbridge UB8 3PH, United Kingdom; § School of Biological and Chemical Sciences, Queen Mary University of London, London E1 4NS, United Kingdom; ∥ The Thomas Young Centre for Theory and Simulation of Materials, London SW7 2AZ, United Kingdom

## Abstract

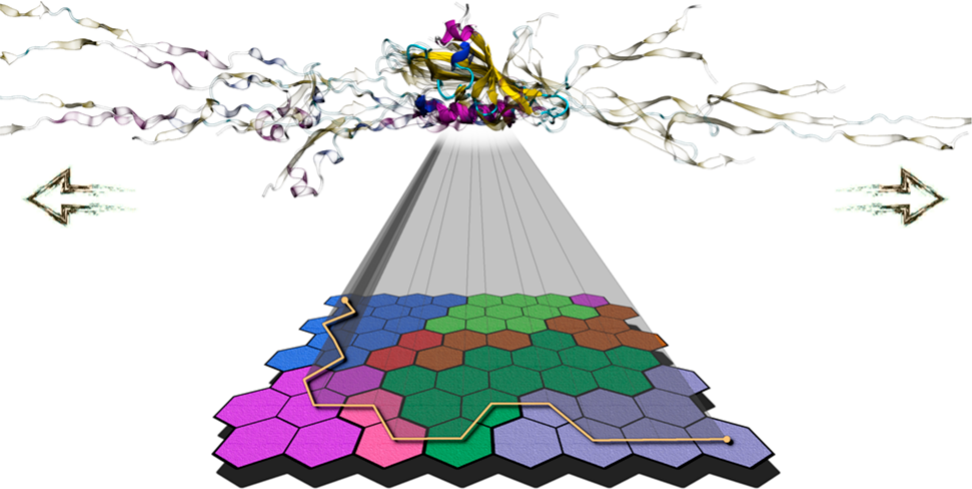

Several experimental studies indicated that large conformational changes, including partial domain unfolding, have a role in the functional mechanisms of the basic helix loop helix Per/ARNT/SIM (bHLH-PAS) transcription factors. Recently, single-molecule atomic force microscopy (AFM) revealed two distinct pathways for the mechanical unfolding of the ARNT PAS-B. In this work we used steered molecular dynamics simulations to gain new insights into this process at an atomistic level. To reconstruct and classify pathways sampled in multiple simulations, we designed an original approach based on the use of self-organizing maps (SOMs). This led us to identify two types of unfolding pathways for the ARNT PAS-B, which are in good agreement with the AFM findings. Analysis of average forces mapped on the SOM revealed a stable conformation of the PAS-B along one pathway, which represents a possible structural model for the intermediate state detected by AFM. The approach here proposed will facilitate the study of other signal transmission mechanisms involving the folding/unfolding of PAS domains.

## Introduction

An increasing number of proteins have been reported showing the ability to switch between different fold arrangements. During their life cycle, these proteins can undergo large conformational changes from an ordered state to an alternative one involving secondary structure shifts or exposure of new surfaces. This behavior is often associated with an expanded functional role of the protein that, thanks to the structural changes, can for example modify the interactions with partners.^1,2^

Experimental evidence has suggested that considerable conformational changes, and perhaps even a switch between folded and partially unfolded states of a domain, have a role in the functional mechanisms of the basic helix loop helix Per/ARNT/SIM (bHLH-PAS) family of transcription factors. Members of the bHLH-PAS family have a broad range of functions in developmental and physiological processes, and some are involved in cancer.^3^ The bHLH-PAS proteins generally act as heterodimers that consist of a signal-regulated subunit (for example, the aryl hydrocarbon receptor (AhR), involved in toxin metabolism, and the hypoxia-inducible factor-α (HIFα) proteins contributing to maintenance of cellular oxygen homeostasis) and a more ubiquitous subunit (for example, the aryl hydrocarbon receptor nuclear translocator (ARNT), which participates in both AhR and HIFα mechanisms by dimerizing with them). These proteins exhibit a relatively well-conserved N-terminal domain structure, including the bHLH and the PAS regions. The latter contains two structurally conserved domains: PAS-A, critical for dimerization selectivity, and PAS-B, responsible for sensing diverse exogenous and endogenous signals.

**Figure 1:**
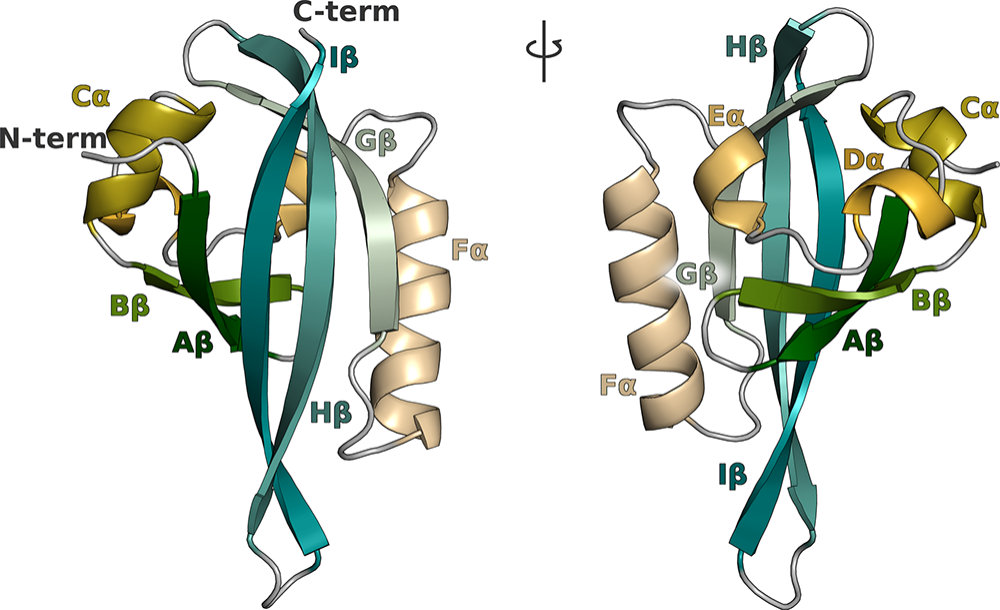
Typical PAS-domain fold is here represented using the three-dimensional structure of the ARNT PAS-B domain (PDBID: 1X0O). Secondary structure elements are a five-stranded antiparallel β-sheet (the N-terminal Aβ, Bβ and the C-terminal Gβ, Hβ, and Iβ) flanked by a long α-helix (Fα, called “helical-connector”) and several shorter α-helices (Cα, Dα, and Eα, often called “helical bundle”).

The PAS domain is present in several proteins also outside the bHLH-PAS family and exhibits a high level of plasticity, fundamental for its role of sensor for different signals, including oxygen, ligands, light, and redox potential.^4^ A typical PAS fold is composed by a central five-stranded antiparallel β-sheet, a long α-helix, and some shorter helices, which surround a buried internal cavity^5^ (Figure 1). An additional N-terminal helix (A′α) was observed in some PAS domains.

In most PAS domains, signals apparently propagate to and through the central β-sheet and ultimately toward spatially remote effector domains, where they modulate biological activity. Signal reception and propagation often involve significant fold changes. In the photoactive yellow protein (PYP), a partial unfolding of the PAS domain was observed upon blue-light absorption.^6,7^ This perturbation causes the dissociation of the A′α helix from the β-sheet surface followed by a conformational change of A′α.^8^ A similar mechanism was also reported for the PAS domains of N. crassa Vivid^9^ and LOV2.^10^ In the sensor histidine kinase (CitA), ligand binding was found to induce a considerable contraction of the PAS domain, followed by partial unfolding of an N-terminal helix.^11^ These similarities suggest that a common signal transduction mechanism involving changes in the β-sheet is conserved among the PAS proteins, despite the wide range of stimuli they sense. The malleability of the β-sheet of the PAS domains is further confirmed by the β-strand slippage observed for PYP in AFM-based pulling experiments.^6^

Evidence of the flexibility of PAS domains has also been found within the bHLH-PAS family, and substantial conformational changes have been suggested to have a role in the function of proteins from this family. Despite the buried nature of the HIF-2α PAS-B cavity, ligand binding was found to occur with rapid association rates, typical of solvent accessible ligand binding sites rather than internal cavities.^12^ The comparable rate constants for compounds with different chemical properties and size suggested a common energetic barrier to overcome, which could be associated with the HIF2α PAS-B conversion from a “closed” structure to a binding-competent “open” state.^12^ Similarly, the AhR PAS-B was suggested to have a more open cavity when associated with the HSP90 chaperone protein and undergoing a substantial conformational change upon ligand binding and HSP90 displacement.^13^ The hypothesis that the open conformation of the AhR PAS-B displays partially unfolded Aβ and Bβ strands was supported by mutagenesis and coimmunoprecipitation experiments showing that a set of residues in these sheets are involved in both HSP90 association and ligand binding (suggesting that they are exposed in the open state and buried into the binding cavity in the closed one).^14^ In addition, other HSP90 clients have been shown to undergo an unfolding of two beta strands to allow chaperone recognition.^15^ Particularly relevant in this framework is the experimental evidence regarding ARNT. Several studies evidenced the remarkable flexibility of the ARNT PAS-B β-sheet. In a recent study, a single-site mutant in this β-sheet was shown to populate an alternative conformation with a three-residue register shift in the Iβ,^16^ and it was observed that the interconversion between the two states proceeds through a chiefly unfolded transition state.^17^ Moreover, single-molecule atomic force microscopy (AFM), used to investigate the mechanical unfolding of the ARNT PAS-B domain, revealed two distinct pathways via a kinetic partitioning mechanism.^18^ A simple two-state pathway was observed for the majority of the unfolding events (67%), whereas in the other 33% of cases a well-defined intermediate state was found in which the C-terminal β-hairpin is detached from the domain. It was suggested that the observed low mechanical stability of the PAS-B domain may help PAS proteins to recruit protein partners and lower the free-energy barrier for the formation of the binding interface.

In this work we use steered molecular dynamics (SMD) simulations to investigate the unfolding process of the ARNT PAS-B domain, with the aim of gaining insight into the involvement of the PAS-domain unfolding in the signal transmission mechanisms of the bHLH-PAS family. This system represents an ideal starting point for our studies given the availability of AFM experiments^18^ for a direct validation of computational results.

SMD applies external forces to manipulate biomolecules in order to probe mechanical functions, as well as to accelerate processes that are otherwise too slow to simulate. This method complements single-molecule AFM experiments and provides invaluable insights into mechanical unfolding processes at an atomistic level.^19−22^ Multiple SMD replicas can be run to sample different unfolding events.^23−25^ The analysis of the resulting trajectories may lead to the discovery of different pathways for the process under study,^26^ but the interpretation of the results is not trivial. Often visual inspection of the trajectories may highlight the conformational differences in the sampled states, but there are no standard protocols to detect and classify multiple pathways. In this work, we developed an original approach based on the use of a self-organizing map^27^ (SOM) to identify different pathways in the unfolding of the ARNT PAS-B. A SOM is considered a type of artificial neural network, with an explicit visual representation of data on a two-dimensional map, which has been widely used for the analysis of different types of data,^28−30^ including protein conformations extracted from MD simulations.^27,32^ Here, we applied a SOM-based approach not only to detect the different conformational states observed during the unfolding but also to reconstruct and classify the unfolding pathways sampled by the SMD replicas. Using this strategy, we were able to identify two different types of unfolding pathways for the ARNT PAS-B domain, which are in good agreement with the available AFM data, as well as to indicate a possible atomistic model for the intermediate state revealed by the AFM experiments in one of the pathways.

## Methods

### Steered MD Simulations

The structure of the ARNT PAS-B domain was downloaded from the Protein Data Bank^33^ (PDBID: 1X0O^34^). The structure was preprocessed for simulation with the Schrodinger’s Protein Preparation Wizard tool,^35^ and residue protonation states were determined by PROPKA^36^ at pH = 7.0. The system was then solvated in a triclinic box with TIP3P water molecules (size of the box: 45 × 8 × 8 nm). The size of the box was set to accommodate the extended ARNT PAS-B polypeptide (40 nm long) on the *x*-dimension. No counterions were added since the system was already neutral. Simulations were run using GROMACS 2018.3^37^ with the Amber ff14SB force field.^38^ A multistage equilibration protocol, as described in ref (39), was applied to remove unfavorable contacts and provide a reliable starting point for the SMD runs: the system was first subjected to 2000 steps of steepest descent energy minimization, with positional restraints (2000 kJ mol^–1^ nm^–2^) on all resolved atoms. Subsequently a 200 ps NVT MD simulation was used to heat the system from 0 to 100 K with restraints lowered to 400 kJ mol^–1^ nm^–2^, and then the system was heated to 300 K in 400 ps during a NPT simulation with further lowered restraint (200 kJ mol^–1^ nm^–2^). Finally, the system was equilibrated during an NPT simulation for 2 ns with backbone restraints lowered to 50 kJ mol^–1^ nm^–2^. In the NVT simulations the temperature was controlled by the Berendsen thermostat^40^ with a coupling constant of 0.2 ps, while in the NPT simulations the V-rescale thermostat^41^ (coupling constant of 0.1 ps) was used and the pressure was set to 1 bar with the Parrinello–Rahman barostat^42^ (coupling constant of 2 ps). A time step of 2.0 fs was used, together with the LINCS^43^ algorithm, to constrain all the bonds. The particle mesh Ewald method^44^ was used to treat the long-range electrostatic interactions with the cutoff distance set at 12 Å. Short-range repulsive and attractive dispersion interactions were simultaneously described by a Lennard-Jones potential, with a cutoff at 12 Å, applying long-range dispersion corrections for energy and pressure.^45^

With structures properly equilibrated, SMD simulations^22^ were performed by harmonically restraining the *x*-component of the distance between the center of mass of the first and last four residues of the protein backbone. A force constant of 500 kJ mol^–1^ nm^–2^ was used, and the equilibrium value of the distance was changed from the initial to the final value at a constant velocity (0.2 nm ns^–1^). The system was simulated for 200 ns to steer the protein to a fully extended conformation. In order to assess the reproducibility of the unfolding pathways, the SMD simulations were run in 50 replicas, for a total simulation time of 10 μs. The force applied to the harmonic spring was monitored during each replica. To test the dependence of the results from the pulling speed, 10 replicas of 1200 ns at a lower velocity (0.02 nm ns^–1^) were also performed, for a total simulation time of 12 μs.

H-bonds were computed using Chimera-X^46^ software with default values for geometrical parameters.

Per-residue nonbonded energy decomposition analysis was performed using the gmx energy command. Pairwise nonbonded contributions of each residue were then summed up to obtain the cumulative interaction energy of each residue with the remaining ones.

### Self-Organizing Maps

A SOM^47,48^ is considered an unsupervised artificial neural network where neurons are arranged in a grid map enforcing topological relationships. Multidimensional input data can be effectively visualized in a low-dimensional representation using a SOM. Each neuron is a feature vector with the same dimension of the input data vectors. Training of the map is an iterative process in which the following are true:

1. The map is initialized with random values for the neuron vectors;
2. Input data vectors are assigned to the neuron with closer feature values, also called the best matching unit (BMU);
3. The feature values of the winning neuron and its neighbors are adjusted toward the values in the input vector. The magnitude of the modification decreases with the distance from the BMU and along the training.

The resulting SOM can be interpreted as an approximation of the data space where similar samples are mapped close together. In this work we used an 8 × 8 sheet-shaped SOM (without periodicity across the boundaries) with hexagonal lattice shape. The input features to train the SOM were selected from the set of pairwise distances among Cβ atoms. Only distances between Cβ atoms closer than 1.0 nm in the native folded conformation were included. This choice was motivated by the evidence that the Cβ contact matrix with 1.0 nm cutoff is within the optimal range for accurate reconstruction of a protein conformation from a set of pairwise atomic distances.^49^ In the native structure of ARNT there are 839 Cβ distances below 1.0 nm. This set of distances was used to build the data set of SMD conformations for SOM training. Input conformations for the SOM training were taken every 100 ps from the SMD simulations at 0.2 nm ns^−1^ pulling speed. In a second step, the neurons are further grouped in a small, but representative, number of clusters by agglomerative hierarchical clustering using Euclidean distance and complete linkage. The optimal number of clusters, *N*, was selected based on the Silhouette profiles. In the present case, selecting the clustering with *N* = 8 produced less neurons with negative silhouette scores compared to other clustering methods with comparable average silhouette width. All the analyses were performed in the R statistical environment^50^ using the *kohonen* package.^51^

### Mapping of Pathways on the SOM

For each SMD simulation, the unfolding pathway was mapped on the SOM by monitoring the BMU of each frame of the simulation and tracking the route covered on the map. The resulting SOM pathways were clustered by agglomerative hierarchical clustering using average linkage. The distance between the SOM pathway of simulation A and that of simulation B is defined as
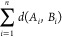
where *n* is the number of frames of each simulation and *d*(*A*_*i*_, *B*_*i*_) is the distance between the BMUs of frame *i* in the two simulations. The distance between two BMUs is defined as the mean square deviation of the two BMU vectors.

SOM can also provide a higher-level representation of the dynamics in the form of a state graph, where important steps in the process are represented by discrete nodes connected according to transition probabilities. To build this graph, an approximate transition matrix between neurons was estimated from the counts of transitions between the starting neuron A and the ending neuron B in all the simulations and repeated for all the possible combinations of A and B. The matrix was then transformed into a row stochastic matrix (dividing each element by the sum of the row). A graph was then built with nodes represented by neurons and edges set to the negative logarithm of the transition probability between the corresponding neurons. The distance between two nodes in the graph was calculated for their shortest path. The distance value was calculated as the negative logarithm of the product of the pairwise transition probabilities between neurons along the path.

Simulations run at 0.02 nm ns^−1^ pulling speed were retraced on the SOM trained with conformations at 0.2 nm ns^−1^ pulling speed, where each frame of the simulations was assigned to the closest neuron. A single SOM was used to analyze both sets of trajectories for an easier comparison and to avoid changes in map topology. We verified that the SOM derived from the highest speed simulations is consistent with the low-speed ones by calculating the distances between the two sets of frames and the closest neurons (Figure S1). Comparable distance distributions were observed for the two sets of simulations, indicating that the low-speed frames are well represented by the SOM even if they were not used for training. All the analyses were performed in the R statistical environment^50^ using the *igraph* package.^52^

## Results

### Mapping Conformations Sampled by Steered MD on SOM

To study the unfolding pathway of ARNT PAS-B under mechanical forces, we performed multiple replicas of SMD simulations using the backbone atoms of the last four residues at the N-terminal and C-terminal ends as pulling groups (see Methods). The simulations were run until the protein was extended at 40 nm, consistent with the extension of the fully elongated polypeptide.^18^

**Figure 2:**
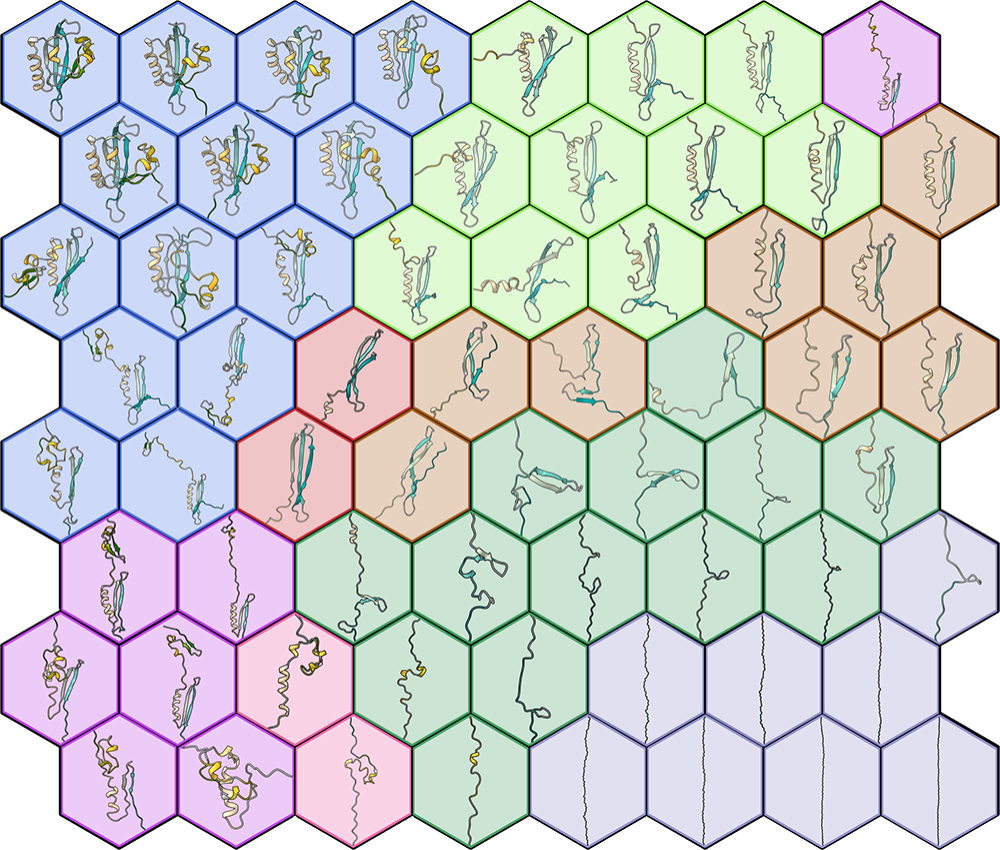
3D structures of the neuron centrotypes represented on the SOM. Colors assigned to neurons refer to cluster analysis that is treated later in the text.

To reconstruct the possible paths of unfolding sampled by different replicas, we used a self-organizing map (SOM), a specific architecture of artificial neural networks, consisting of a grid of neurons (hexagons of the map). The map was trained using protein conformations from the steered MD simulations. After training, each input conformation is assigned to a single neuron so that similar conformations are represented by the same neuron, and similar neurons are close to each other. In this work, a square 8 × 8 SOM without periodic boundaries conditions was used with the distances among Cβ atoms as neuron features (see Methods). Different SOM parameters (SOM shape and neuron features) were tested to obtain an efficient grouping of the protein conformations on the map (data not shown). Figure 2 represents the three-dimensional structures of the centrotypes of each neuron (conformation closest to the neuron vector).

In the trained map, the completely folded and completely unfolded conformations (first and last frames of the SMD simulations) are at the top left and bottom right corners of the map, respectively. Intermediate conformations along the unfolding path populate the other regions of the map.

**Figure 3:**
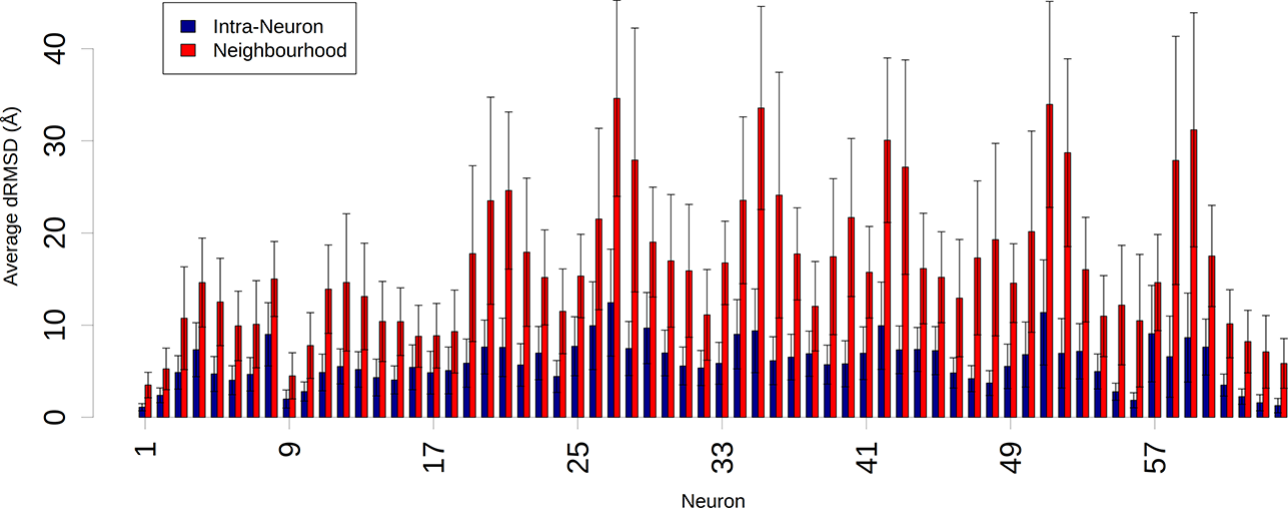
Distribution of dRMSD values calculated between structures from the same neuron (blue) and between structures assigned to neighboring neurons (red). The neurons numbering is shown in Figure S2.

To evaluate the quality of the method in separating different conformations, we compared the distance root mean square deviation (dRMSD) values calculated between structures from the same neuron (blue in Figure 3) with the values calculated between structures belonging to neighboring neurons (red). The intraneuron differences are significantly smaller, indicating an effective segregation of similar conformations in each neuron.

**Figure 4:**
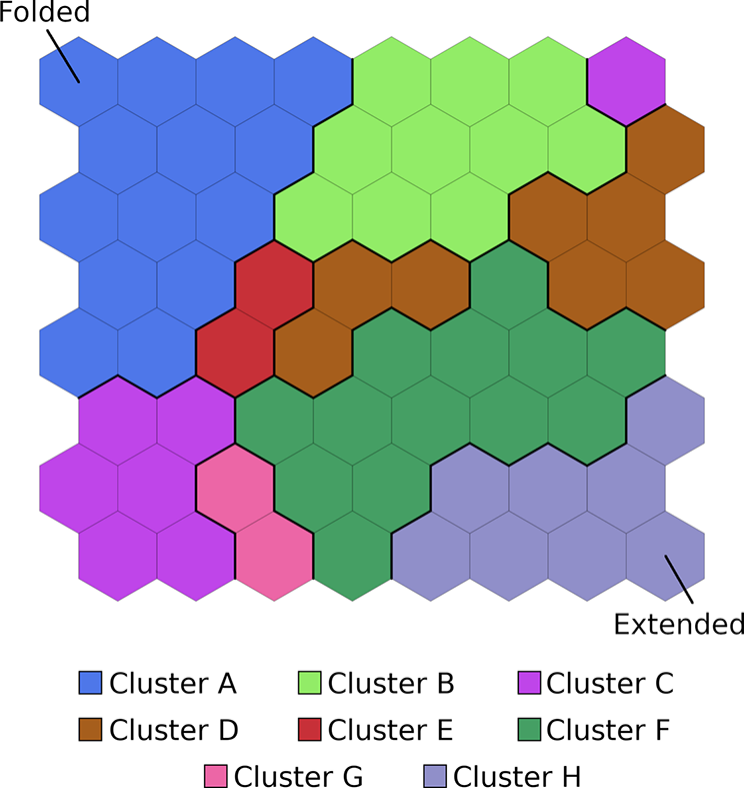
Self-organizing map. Conformations sampled in the different replicas are first assigned to different neurons (tiles of the map), and then neurons are further grouped by hierarchical clustering (colors of the tiles). See Methods for a detailed description.

To obtain a more “coarse-grained” view of the process and highlight putative macrostates sampled during the unfolding, the neurons were further grouped by agglomerative hierarchical clustering (clusters A–H in Figure 4). An optimal number of eight clusters was selected based on the analysis of the silhouette profiles (Figure S3) and the visual inspection of the resulting clusters.

A diagram of the secondary structure composition of frames within each neuron is reported in Figure S4. The average composition in secondary structure and the radius of gyration for each cluster are reported in Table S1.

In addition to canonical descriptors of secondary structures, annotations using structural alphabets are particularly effective in capturing local conformations and their dynamical changes.^53^ Local structures from the ensembles in each neuron were encoded with a structural alphabet (SA),^54^ and a per-fragment profile of divergence from the distribution of local conformations in the folded state was calculated (see the Supporting Information). These profiles were mapped on each of the associated SOM neurons (Figure S5). The analysis highlighted local patterns of unfolding in intermediate neurons; in particular, clusters B and E show changes in conformational dynamics mainly at the N-terminal fragments of the protein, and cluster C does so more frequently at the C-terminal region of the protein.

### SOM Highlights Different Unfolding Pathways

**Figure 5:**
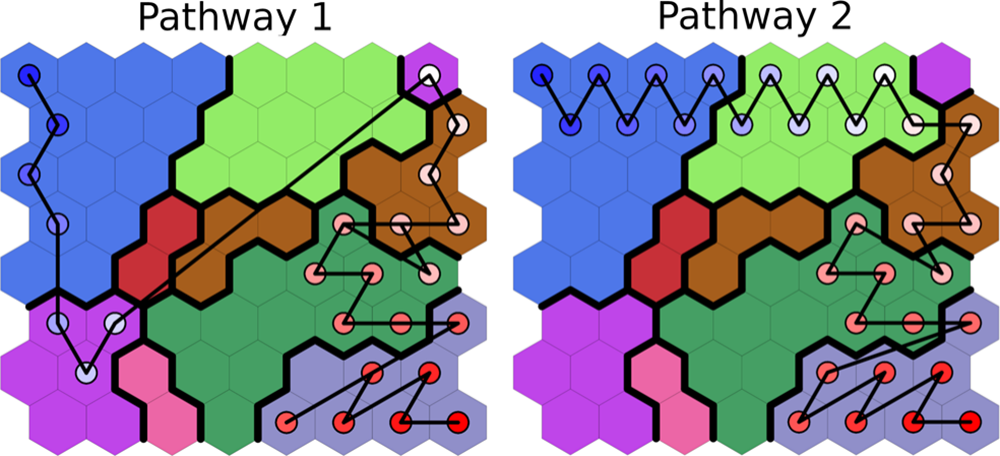
Tracing of two replicas representative of pathways 1 and 2 on the SOM.

In order to analyze the unfolding pathways followed during the simulations, we mapped each trajectory onto the SOM by tracking the position of each frame on the map. All simulations started from the upper left corner and ended in the lower right corner, but we classified the simulations into two distinct types of pathways: pathway 1, visiting neurons in the lower left corner of the map (left in Figure 5), and pathway 2, going through neurons in the top right corner of the map (right in Figure 5).

The mapping of each trajectory replica is shown in Figures S6 and S7. Pathway 2 was the most probable and was sampled in 74% of the replicas.

The above classification was assessed performing hierarchical clustering of the simulation pathways (using average linkage, as detailed in Methods) and analyzing the resulting dendrogram (Figure S8). This diagram mostly agrees with our classification except for the identification of two additional minor pathways (pathway 1b for replicas 1, 26, 42 and pathway 2b for replicas 8, 35, 39) that are grouped in different small branches of the dendrogram. We grouped replicas of pathway 1b to pathway 1, and replicas of pathway 2b to pathway 2, for their similarity to the characteristics of each pathway in the first part of the unfolding process.

Simulations following either pathway 1 or 2 visit most of the eight clusters on the SOM. Two clusters remain exclusive and differentiate the type of path: cluster B was only sampled by simulations following pathway 2, while cluster C was only sampled by simulations following pathway 1. Interestingly also cluster E was only sampled by replicas 8, 35, and 39 and cluster G by replicas 1, 26, and 42. The transition of these replicas through the E or G clusters explains their assignment to separate branches of the dendrogram in the hierarchical clustering of the simulation pathways.

**Figure 6:**
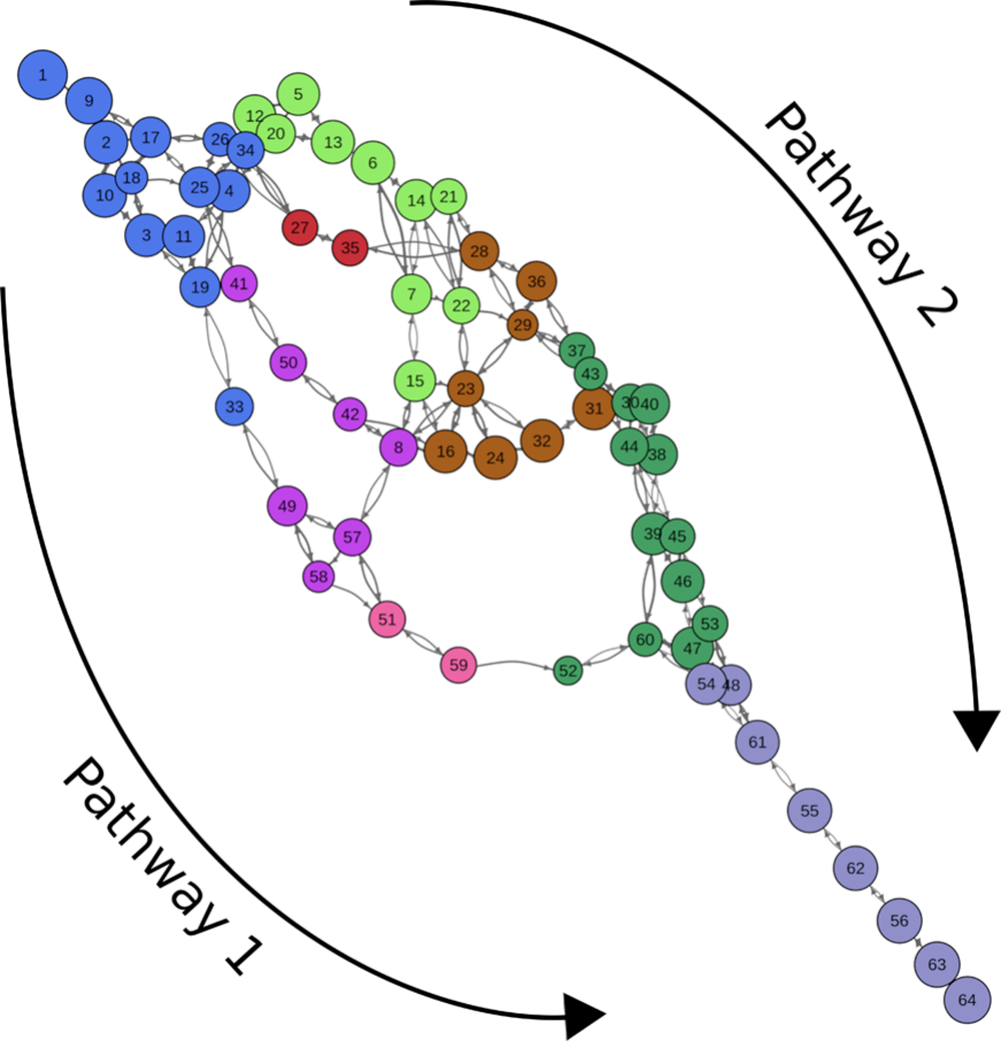
Graph representation of the transition matrix for the SMD unfolding of the PAS-B domain. Nodes are colored according to the cluster colors of Figure 2.

From pairwise transitions between neurons, we reconstructed an approximate transition matrix. This matrix was then visualized as a graph in which nodes represent the SOM neurons, and the edges are weighted by the negative logarithm of the transition probability between pairs of neurons (Figure 6). The sum of edge weights along a pathway is proportional to the logarithm of the combined probability along that pathway (see the Methods section). The two unfolding pathways are clearly visible in this representation.

**Figure 7:**
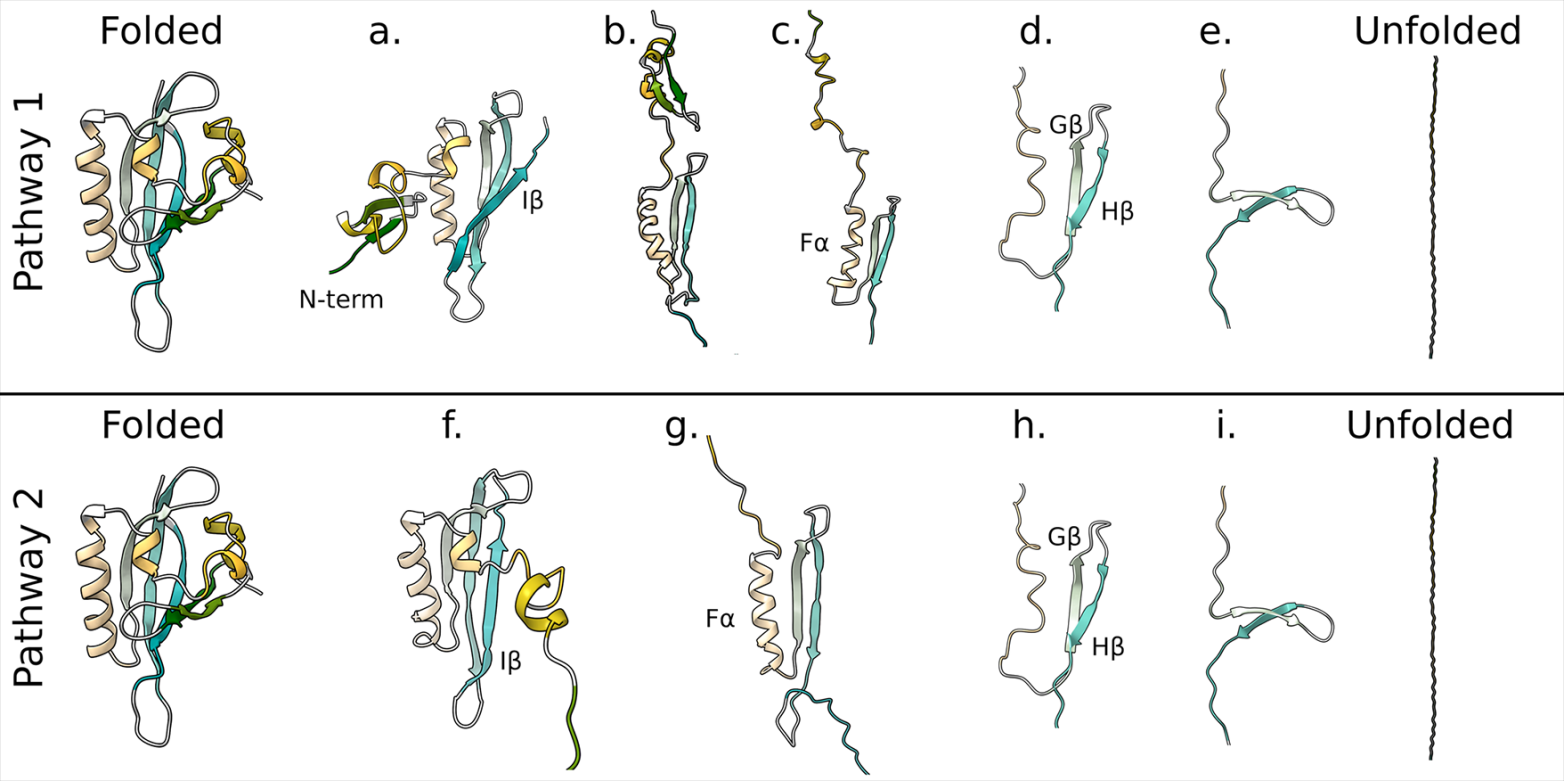
Three-dimensional representation of unfolding pathways. Pathway 1 starts with the detachment of the N-terminal region (a), then unfolding of Iβ (b), elongation of the N-terminal region (c), unfolding of Fα (d), and finally unfolding of Gβ and Hβ (e). Pathway 2 starts with detachment and unfolding of the N-terminal region (f) before the unfolding of the Iβ (g). Then the pathway proceeds as in the case of pathway 1 (h and i). Secondary structure colors are consistent with Figure 1.

Analysis of the structural changes for the different pathways indicates that simulations following pathway 1 start with the detachment of the N-terminal region (Aβ, Bβ, and helical bundle) from the rest of the protein (a in Figure 7). This region, however, preserves its internal contacts and does not completely unfold until the C-terminal Iβ is completely unfolded (b). Then the simulations continue with the unfolding of either the N-terminal region (most of pathway 1 replicas) or the C-terminal region (only in replicas 1, 26, and 42 that visit cluster F). Pathway 2 differs from pathway 1 in the order of events leading to the Iβ unfolding: while in pathway 1 the N-terminal region detaches from the core of the protein but remains “folded”, in pathway 2 the N-terminal region completely unfolds (f in Figure 7) before the Iβ (g).

The different timing for the unfolding of Iβ is also evident from the time evolution of protein backbone H-bonds: Figure S9 reports the extension of the protein corresponding to the last frame where each H-bond was detected in the different replicas. In simulations following pathway 1, the H-bonds between Hβ and Iβ (dark blue) break in the first part of the simulation (before the extension of the protein reaches 10.5 nm), while H-bonds in the N-terminal part (Cα (dark green) and bundle-Bβ (orange)) break at a later time. An opposite behavior is observed in pathway 2 simulations, where the N-terminal H-bonds break in the first part of the simulation and the Hβ-Iβ ones break in the second part. This is consistent with the AFM results that showed an intermediate with Iβ unfolded before 10.5 nm in one-third of the experiments.^18^

**Figure 8:**
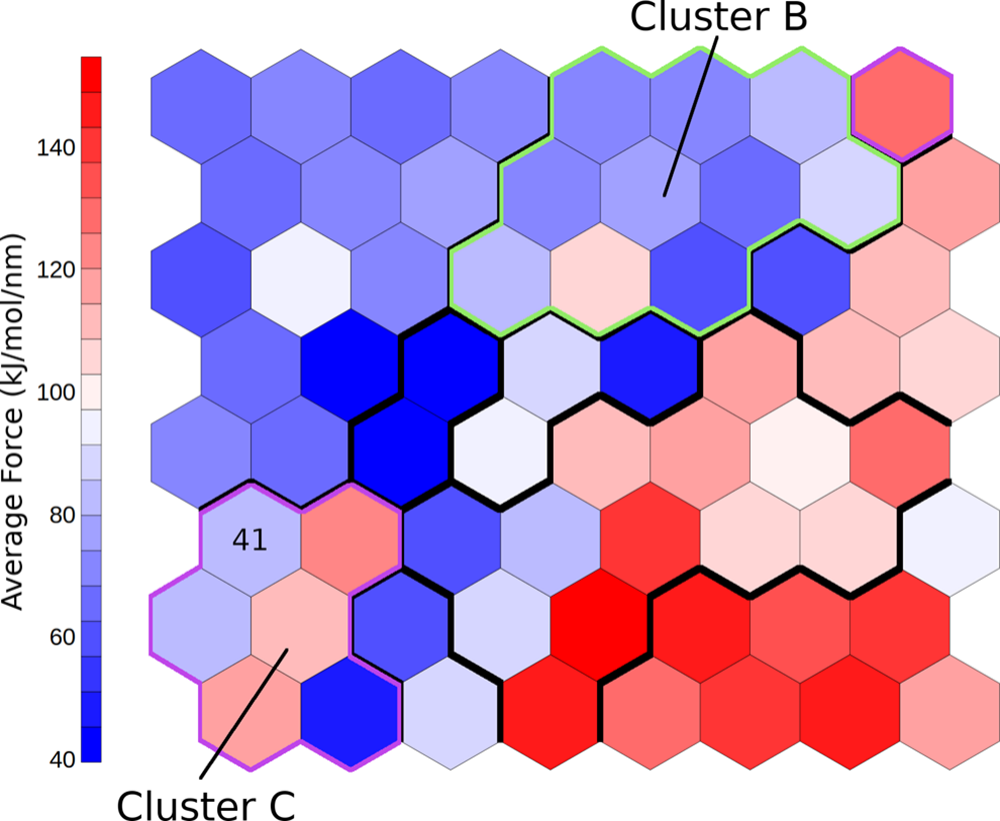
Average SMD forces mapped on the SOM. Neuron 41, discussed in text, is labeled. Cluster boundaries are highlighted for cluster B (green) and C (magenta).

To assess the presence of an unfolding barrier along one of the two pathways, we mapped the SMD forces on the SOM by calculating the average force over the frames belonging to each neuron (Figure 8). Most of the frames belonging to cluster C (sampled only by pathway 1 trajectories) are associated with higher forces compared to the adjacent clusters, which could be interpreted as the presence of a force peak consistent across different replicas and suggests the presence of an intermediate state just before those frames. This observation led us to consider frames belonging to neuron 41 (within cluster C and often visited before the high-force frames) as an intermediate state along pathway 1. From the analysis of the accessible pathways on the graph representation in Figure 6, it can be noted that neuron 41 is often visited along pathway 1. The only alternative route along pathway 1 is through neuron 49 that however shares fundamental features of neuron 41, like the folding of the helical bundle. The frames in neuron 41 have a length extension (9.4 ± 1.0 nm) consistent with that of the intermediate observed in AFM experiments (10.5 nm). On the contrary, frames belonging to cluster B (exclusive of pathway 2) are associated with low forces, without clear peaks along the pathway, which do not suggest the presence of a stable intermediate conformation along the path.

**Figure 9:**
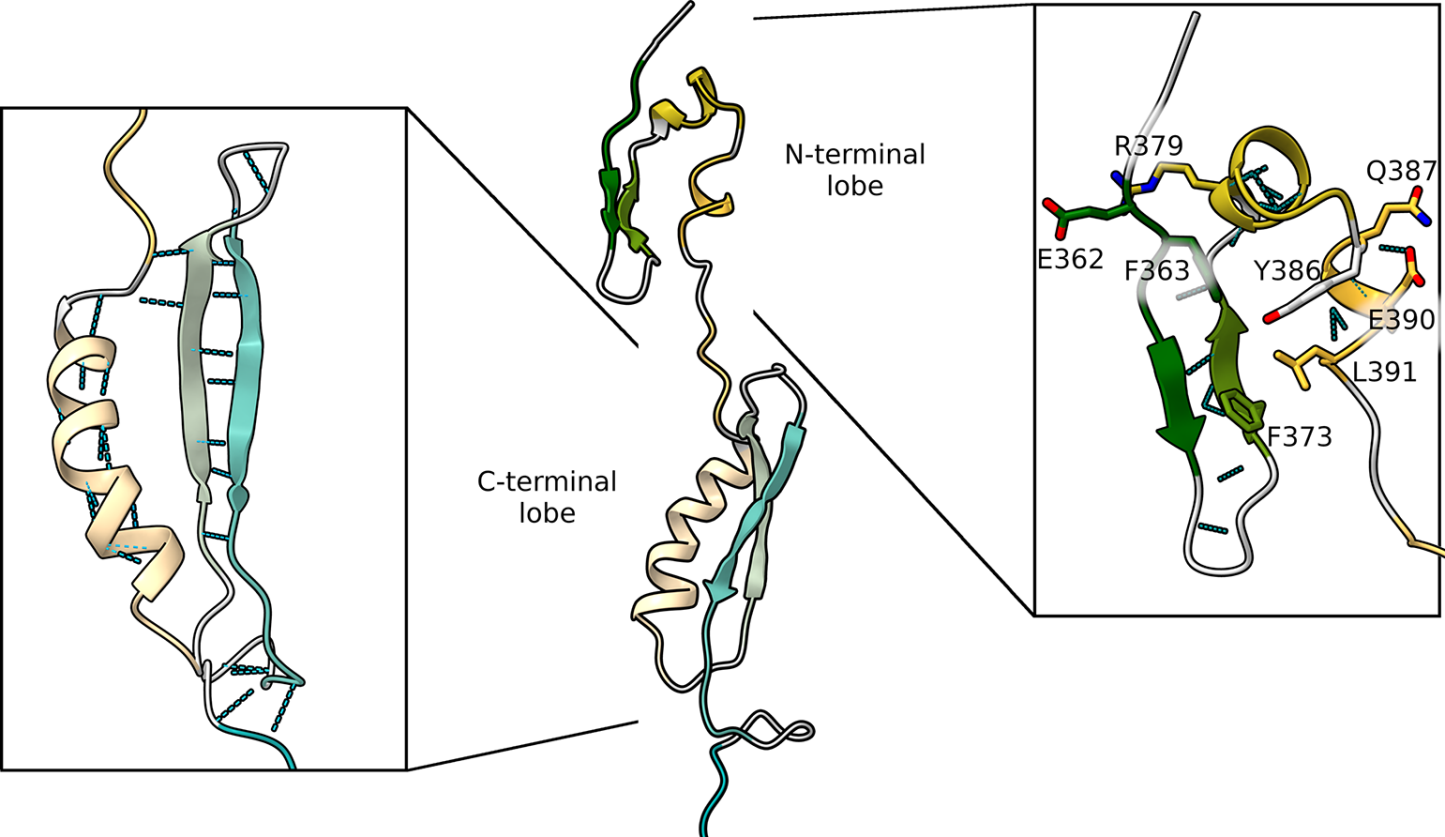
Three-dimensional representation of the putative intermediate state during pathway 1 unfolding. The two insets show the backbone hydrogen bonds in the C-terminal lobe (left) and the group of residues that mostly contribute to stabilizing the N-terminal lobe (right). Secondary structures are colored according to Figure 1.

Conformations belonging to Neuron 41 are highly structured, with the PAS domain split into two lobes: the N-terminal lobe composed of Aβ, Bβ, Cα, and Dα and the C-terminal lobe composed of Fα, Gβ, and Hβ (Figure 9).

Surprisingly, each lobe remained well folded preserving almost all the backbone H-bonds. The N-terminal lobe, in particular, showed great resistance to the unfolding process despite the lower number of native hydrogen bond interactions that stabilize its fold (10 compared to the 22 found in the C-terminal β-strands). Indeed, even more surprising are the simulations passing through cluster F, where the N-terminal lobe remains folded until the complete unfolding of the C-terminal lobe. With the aim of detecting the residues that mostly stabilize the N-terminal lobe in this type of conformation, we performed a per-residue energy decomposition analysis on conformations belonging to neuron 41 (Figure S10). This analysis highlighted the residues that give the most stable interactions: a group of hydrophobic residues (F363, F373, Y386, L391) in the core of the protein and two pairs of residues involved in electrostatic interactions (Glu362-Arg379 and Glu390-Gln387). Together, the above interactions hinder the unfolding process of the N-terminal lobe (Figure 9, right inset).

### Retracing of Low Pulling Speed SMD on the Trained SOM Confirmed the Two Pathways

To verify the effect of the SMD pulling speed on the conformations sampled in the unfolding process, 10 replicas at lower pulling speed were run for 1200 ns, up to an extension of about 24 nm. No alternative pathway was detected aside from the ones already recorded at a faster pulling speed. While these simulations did not cover the full unfolding for time limitations, they were long enough to compare the initial stages of the unfolding in the two sets of replicas.

**Figure 10:**
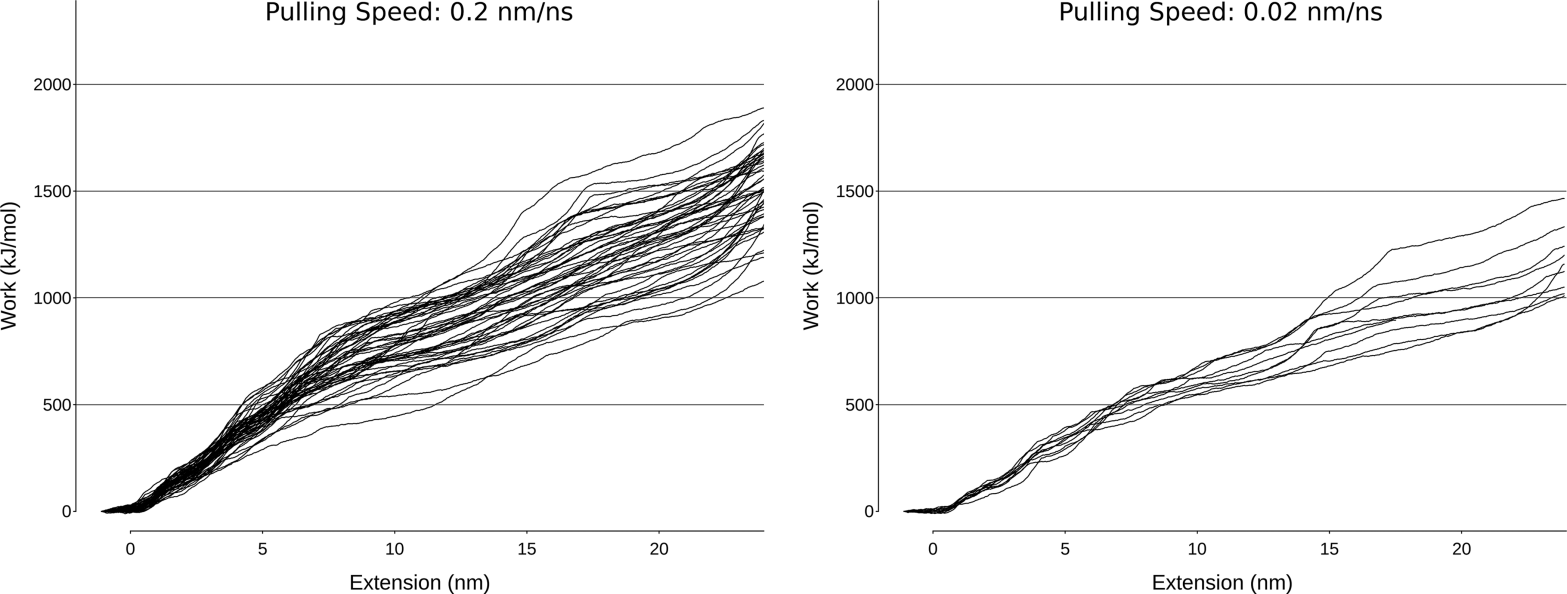
Work profiles for replicas at higher (0.2 nm ns^–1^ on the left) and lower (0.02 nm ns^–1^ on the right) pulling speeds.

The work performed in the 0–24 nm extension range (the region sampled at both pulling speeds and where most of the unfolding process takes place) is reported as a function of the extension length in Figure 10.

Despite the difference in pulling speed, the total work in the low-speed simulations (1000–1500 kJ/mol) falls within the range of the higher speed ones (1000–2000 kJ/mol), indicating that the pulling speed of 0.2 nm ns^–1^ is still able to capture lower energy pathways.

**Figure 11:**
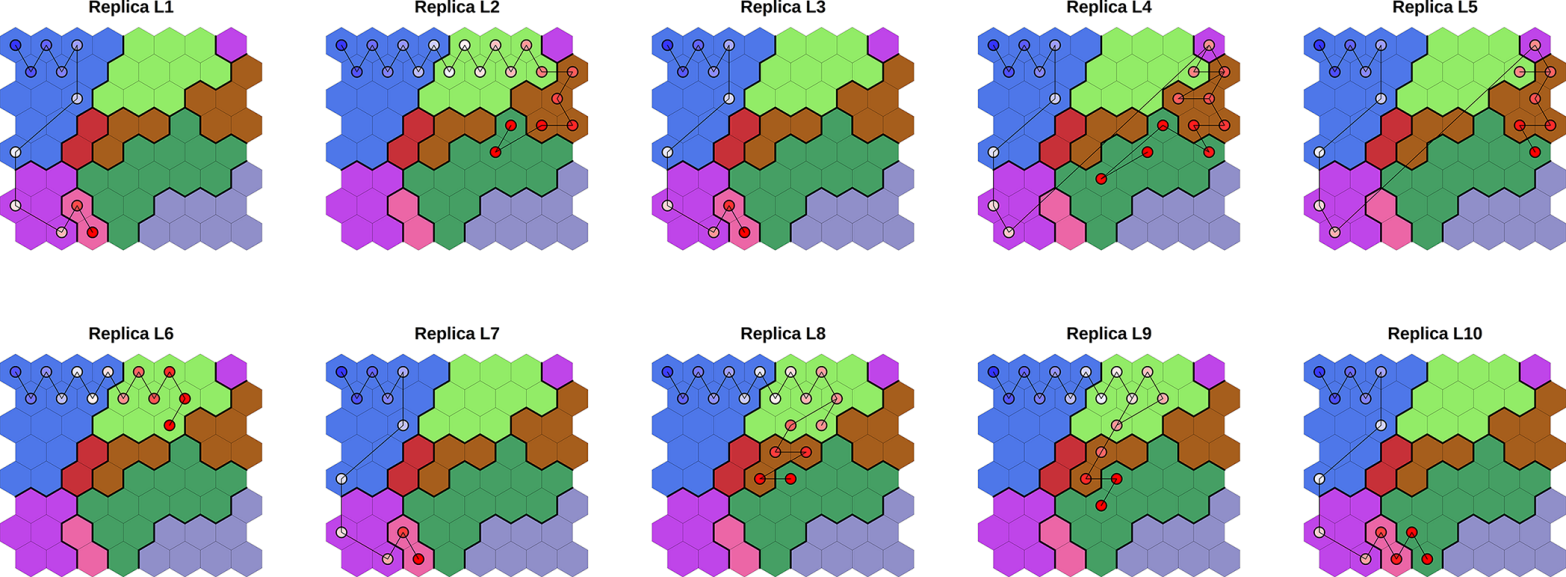
Evolution of low pulling speed (0.02 nm ns^–1^) SMD simulations, plotted over the 0.2 nm ns^−1^ SOM. For each replica, frames were assigned to the nearest neuron and represented as circles colored in a blue–white–red color scale according to their time in the simulation. Consecutive frames are connected by solid lines.

Mapping the low-speed simulations over the SOM (Figure 11) shows that they are very similar to the initial part of the higher speed simulations (Figures S4 and S5). In particular, six low-speed simulations visit cluster C (pathway 1), while the other 4 go through cluster B (pathway 2).

At a lower pulling speed we found a relative difference in the frequency of sampling of the two pathways, but this is expected due to the limited sample (10 replicas). These data provide limited statistics. However, further exploration of the process at low speed would require significant computational resources, and it is beyond the scope of the present work.

## Discussion

In this work we present a novel computational approach to analyze large conformational changes in SMD simulations using SOMs. The approach is applied to the characterization of the mechanical unfolding of the ARNT PAS-B domain.

In SMD, the use of multiple replicas is crucial to sample all the possible unfolding routes. However, this usually leads to a large amount of complex information, which is difficult to translate into a unified and simple representation of the process. A strategy that is often used to extract information from MD trajectories is cluster analysis, which generates a reduced set of nonredundant structures that are representative of the main features of the simulations. A variety of clustering approaches exist, many of which are optimized for the analysis of conformational ensembles of proteins.^27,55,56^ Popular choices include hierarchical clustering, linkage, and k-means.^55^ Among the different solutions, artificial neural networks have emerged as particularly successful for many applications in bioinformatics, chemometrics, and computational chemistry.^27^ SOMs, in particular, are a powerful data-analysis method that combines the advantages of an adaptive learning process with the ability to produce a topological mapping.

In this work, SOMs were used to obtain a geometrical clustering of the ARNT PAS-B conformations. The use of SOM bidimensional visualization facilitates the identification of major conformational states and unfolding pathways.

The use of Cβ atom distances instead of Cartesian coordinates as descriptors of protein conformations made the calculation of the dissimilarity matrix superposition-independent. This removed the effect of structural alignment errors, which can be particularly evident in unfolding simulations due to the large conformational changes. The reference set for superposition was limited to the Cβ distances within 1.0 nm in the native conformation with the advantage that the similarity measure was driven by detecting nativelike contacts and interactions. Finally, the use of a 2D map without periodic boundary conditions automatically segregated the end points of the process (folded and unfolded states) in two corners of the map. These features enhanced the readability of the map, and the final SOM well represented the most important conformational states.

Taking advantage of the topological mapping of the SOMs, in this work we introduced for the first time the idea of tracing the pathways followed by different SMD replicas on the map to obtain an immediate visualization of differences among the sampled pathways. This approach led us to identify two groups of pathways that undergo unfolding of secondary structures in a different order. Pathway 1 starts with a rigid detachment of the N-terminal region, followed by unfolding of the Iβ and subsequently of the N-terminal region, while in pathway 2 the whole N-terminal region unfolds at first, followed by Iβ.

Pulling forces associated with each frame of the simulations are informative of the process, but detecting patterns in the force profiles from the different replicas is difficult due to the different stages in which they reach peak forces. Through analysis of average forces mapped on the SOM, we were able to highlight conformations along pathway 1 with maximum forces, while neurons along pathway 2 are all characterized by lower forces. This suggested the presence of a stable intermediate conformation along pathway 1.

Our findings are in agreement with previous AFM experiments that indicate the existence of two unfolding pathways, with one of them characterized by an intermediate state at about 10.5 nm extension.^18^ Not only did we find a probability distribution for the two pathways (24% pathway 1 vs 76% pathway 2) comparable to the experimental one (33% pathway 1 vs 67% pathway 2), but also the intermediate state identified on neuron 41 has the features highlighted by the experiments, i.e., an extension of about 10 nm and an unfolded Iβ region.^18^ The description of the intermediate state, emerging from our analysis, complemented or added to the experimental findings by offering a possible structural model.

It has been suggested that other proteins in the bHLH-PAS family, acting as transcription factors in key developmental and physiological processes, may exert their functions through major conformational changes within their PAS domains.^12,13^ However, for these proteins no experimental data on the unfolding process and no evidence on the structure of their putative partially unfolded states have been obtained. The approach proposed in this work for simulating the unfolding processes of PAS domains and analyzing the metastable states along the different pathways may contribute to shed light into the role of PAS-domain folding/unfolding events in the signal transmission mechanisms of the bHLH-PAS proteins.

## Supporting Information Available

Analysis of local conformational changes with a Structural Alphabet; Boxplot reporting distances between each frame of simulations and the closest neuron on the SOM; SOM with neuron numbering labeled on each unit; choice of the best number of clusters; secondary structure composition for each neuron; local backbone conformational changes according to Structural Alphabet encoding; trace paths on the trained SOM for simulations following pathway 1 and 2; dendrogram of hierarchical clustering of the pathways followed by different replicas; boxplots reporting the extension of the protein corresponding to the last frame where each native H-bond was recorded in the different replicas; per-residue nonbonded energy from decomposition analysis; average percentage of secondary structure type in structures from each cluster and average radius of gyration for each cluster (PDF)

## Abbreviations

bHLH-PAS: basic helix loop helix Per/ARNT/SIM
AFM: atomic force microscopy
SMD: steered molecular dynamics
SOM: self-organizing map
AhR: aryl hydrocarbon receptor
HIFα: hypoxia-inducible factor-α
ARNT: aryl hydrocarbon receptor nuclear translocator
PYP: photoactive yellow protein
BMU: best matching unit
dRMSD: distance root mean square deviation
