# Supplementary material for: Reconstruction of ARNT PAS-B Unfolding Pathways by Steered Molecular Dynamics and Artificial Neural Networks
Source: J Chem Theory Comput. 2021 Mar 29;17(4):2080–9. doi: 10.1021/acs.jctc.0c01308 (PMC8047803; doi:10.1021/acs.jctc.0c01308)
Supplement: Supplementary file 1 — ct0c01308_si_001.pdf [file ct0c01308_si_001.pdf]

Reconstruction of ARNT PAS-B Unfolding Pathways by Steered Molecular Dynamics and Artificial Neural Networks

Stefano Motta, Alessandro Pandini, Arianna Fornili, Laura Bonati**

Analysis of local conformational changes with a Structural Alphabet

Local conformational changes were analyzed using a discrete state model based on a Structural Alphabet (SA)[1]. The SA selected for this analysis is a collection of 25 representative conformations of 4-residue protein fragments, each labelled with a letter. Each conformation is identified by the geometry of the four C-alpha atoms in the fragment. Protein structures can be encoded into a SA string, progressively labeling each overlapping four-residue fragments with the SA letter that has minimum RMSD among the representative conformations. Structures belonging to each neuron were extracted from the trajectory and encoded with the SA. The ensemble distribution of letters assigned to each fragment position in the sequence was then compared to the reference folded state (neuron 1 in Fig S2) by means of the Kullback-Leibler divergence:

$$\sum_i P(i) \log_2 \left(\frac{P(i)}{Q(i)}\right)$$

where P(i) is the discrete distribution for the fragment *i* in the neuron and Q(i) the discrete distribution for the fragment *i* in the reference state. Following this strategy, we obtained a per-fragment profile of divergence from the folded state. This analysis captures the details regarding the local unfolding in terms of conformational ensembles of local structures and their changes during the process.

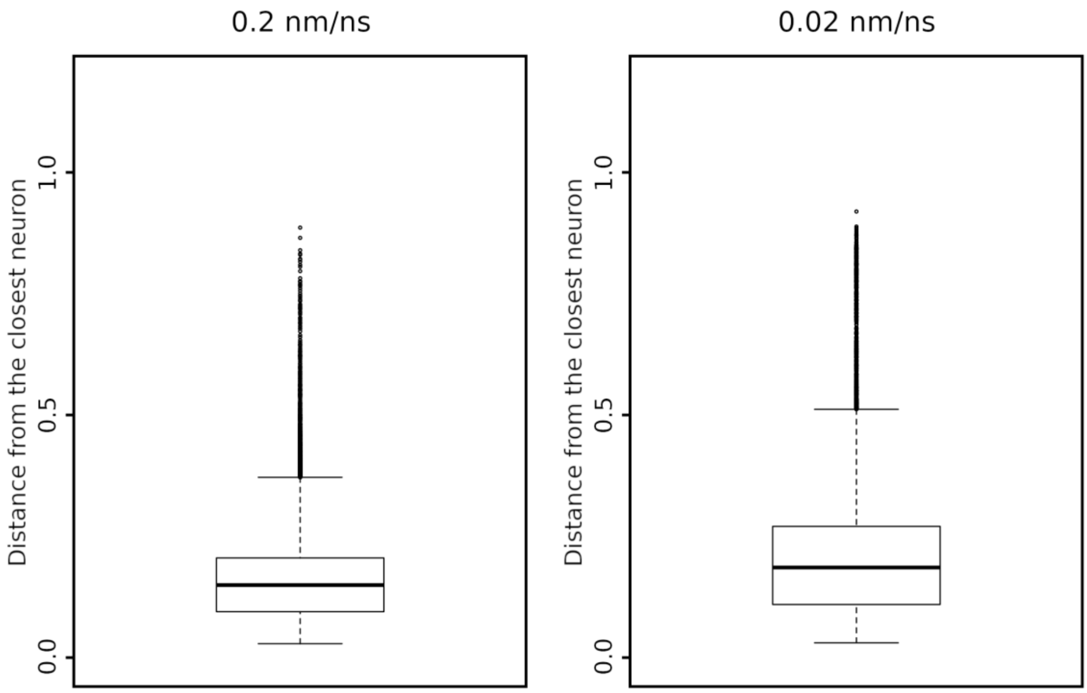

Figure S1: Boxplot reporting distances between each frame of simulations and the closest neuron on the SOM: simulations at 0.2 nm/ns pulling speed (left) and at 0.02 nm/ns pulling speed (right).

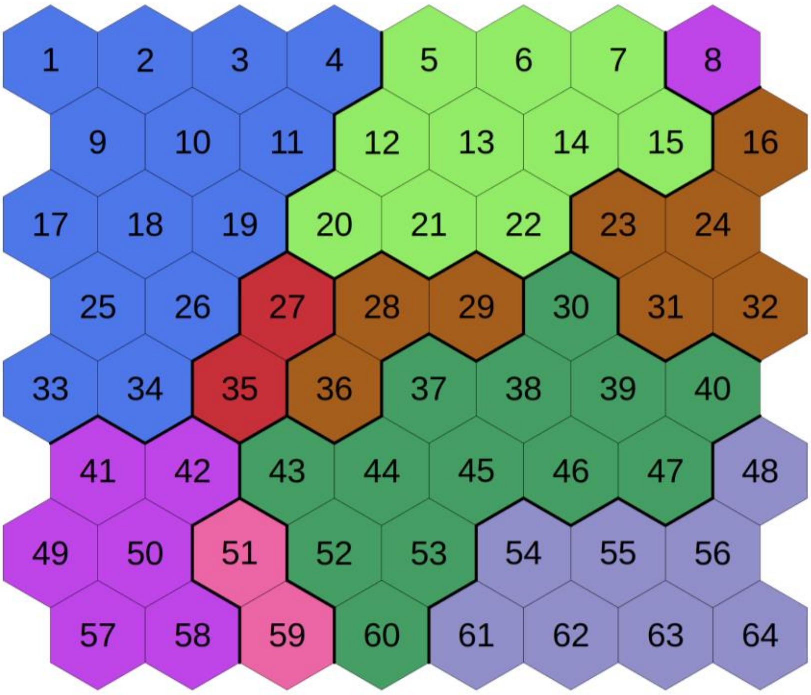

Figure S2: Self-Organizing Map with neuron numbering labelled on each unit.

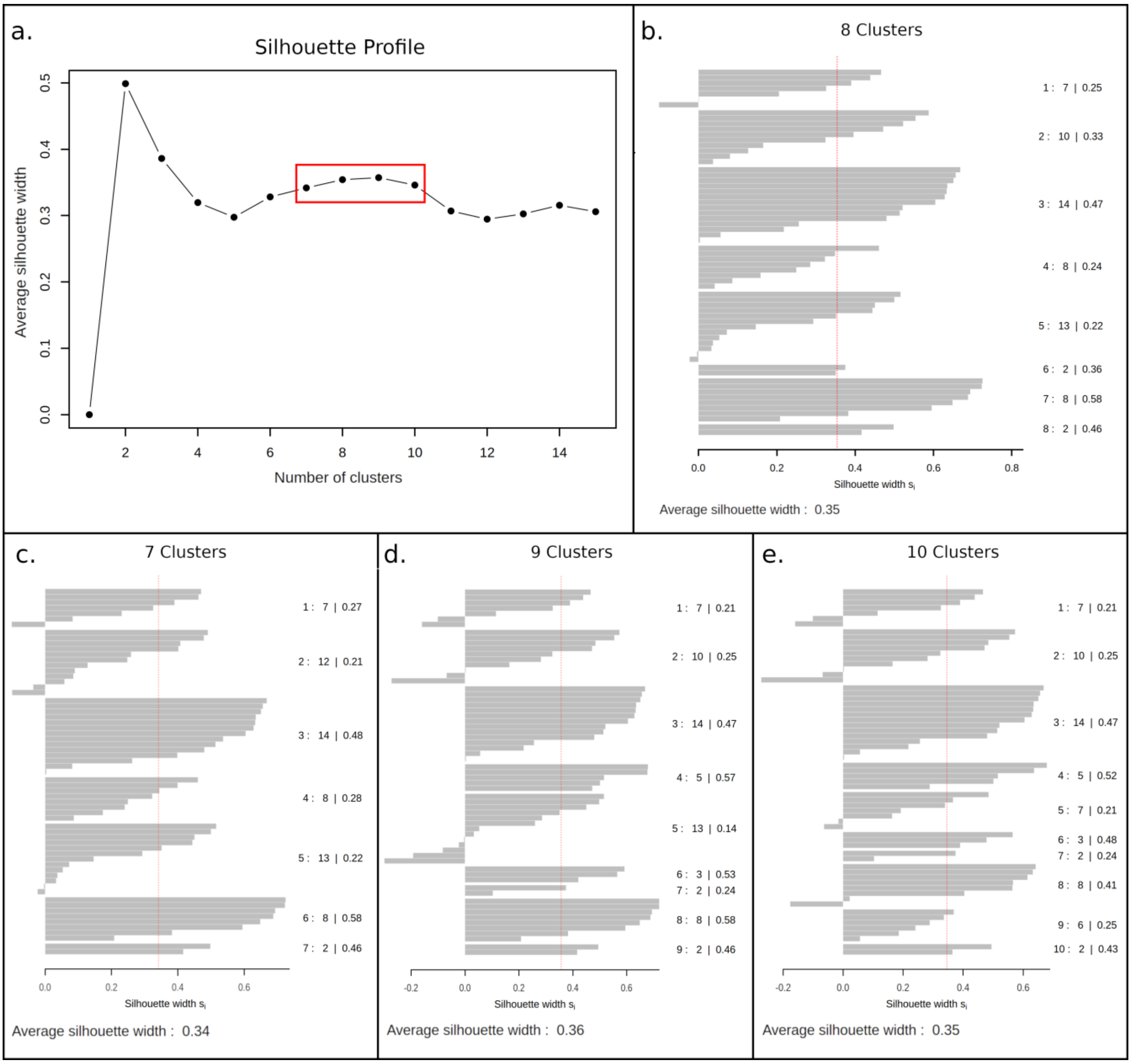

Figure S3: Choice of the best number of clusters. The average silhouette width is reported against the number of clusters (panel a.). For the chosen number of clusters (panel b.) and the other promising choices (panel c. d. e.) the detailed silhouette profiles are reported.

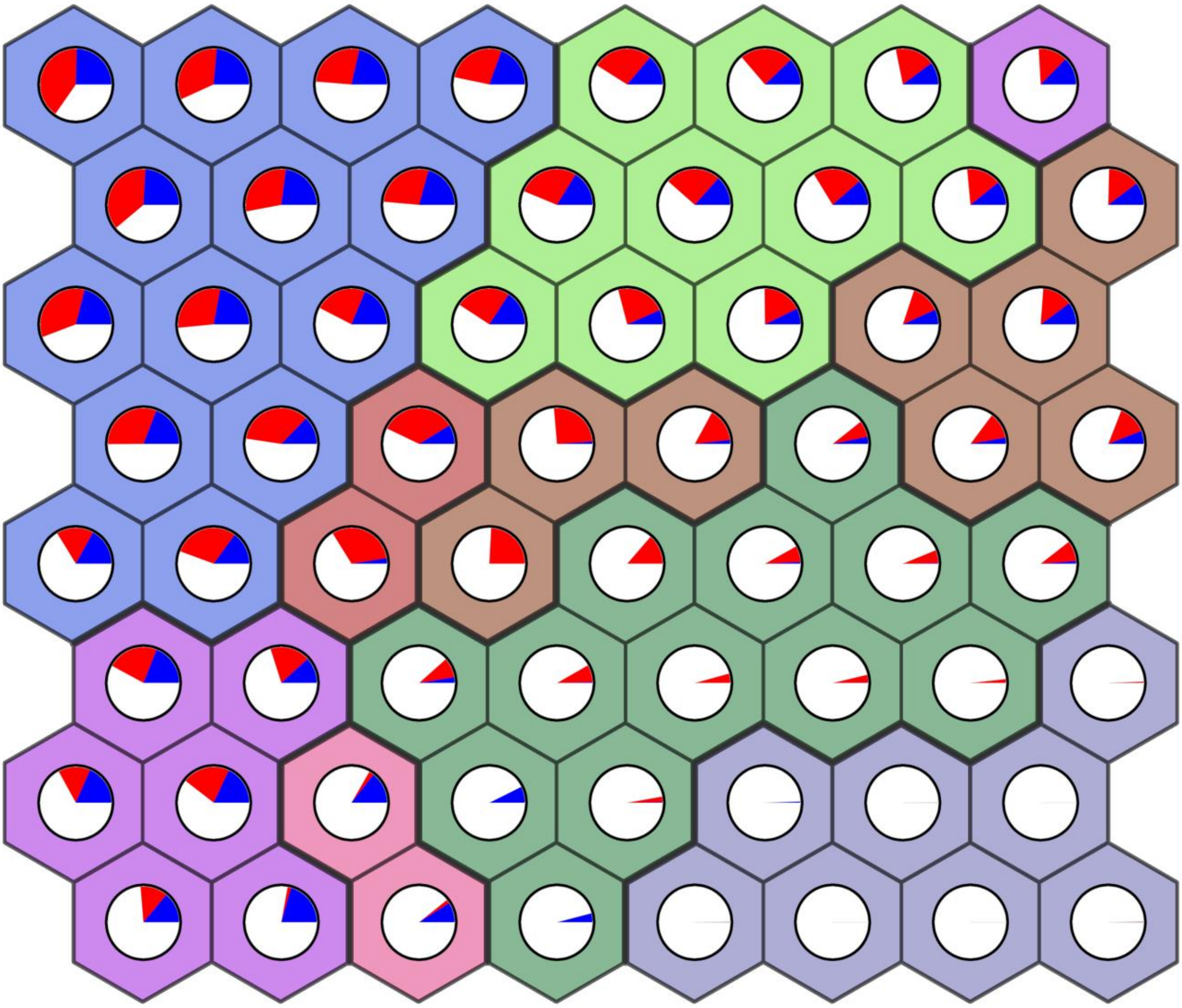

Figure S4: Secondary structure composition for each neuron. Average composition for frames belonging to each neuron was represented as pie-chart with helical structures in blue, β-sheet in red and unstructured segments in white. Secondary structure assignment was performed with dssp[2].

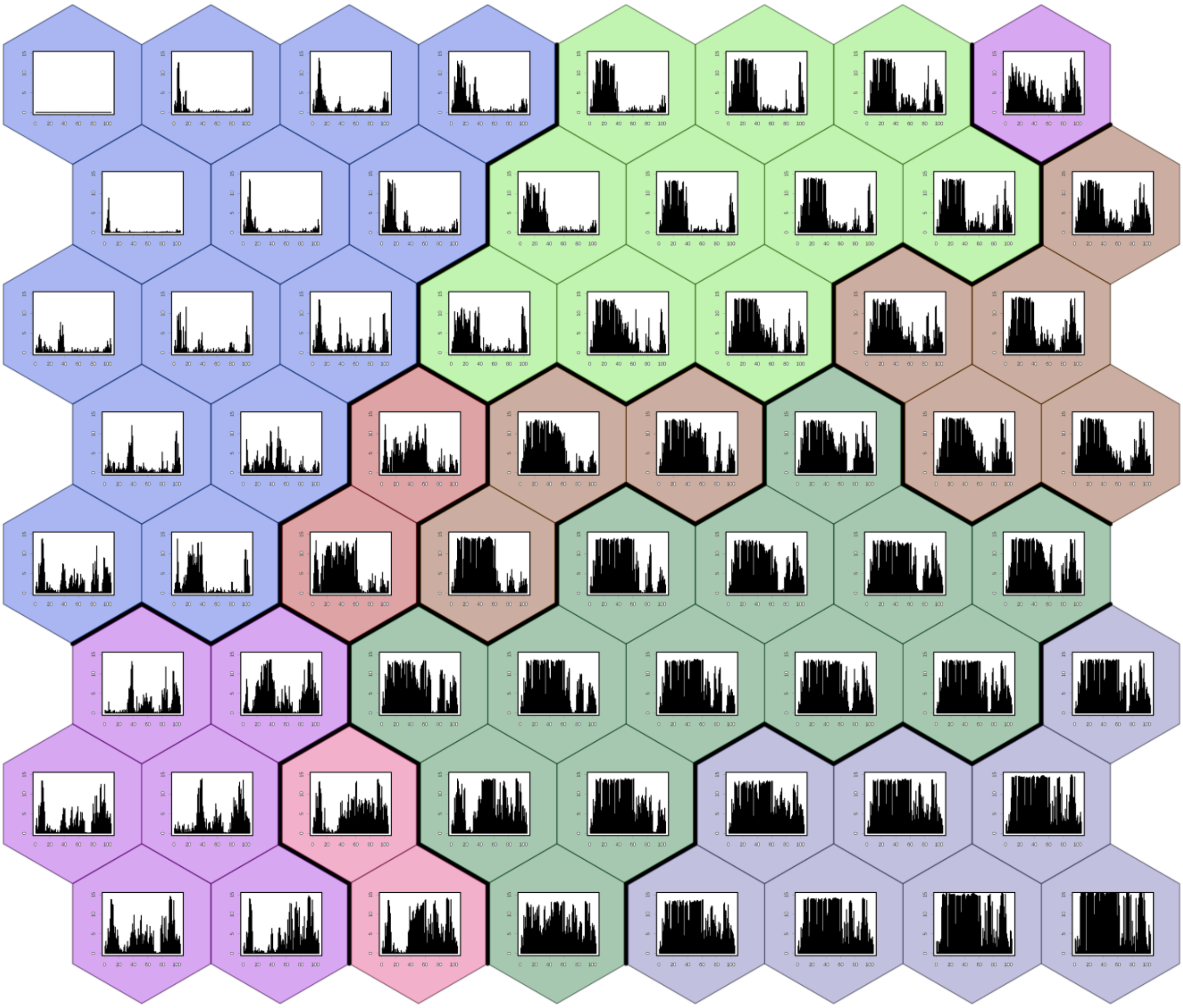

Figure S5: Local backbone conformational according to Structural Alphabet encoding. Changes in ensemble distributions for each backbone fragment are reported for structures belonging to each neuron. The change is expressed as Kullback–Leibler divergence from the reference state in the neuron representing the folded native state (neuron 1 in Fig S2).

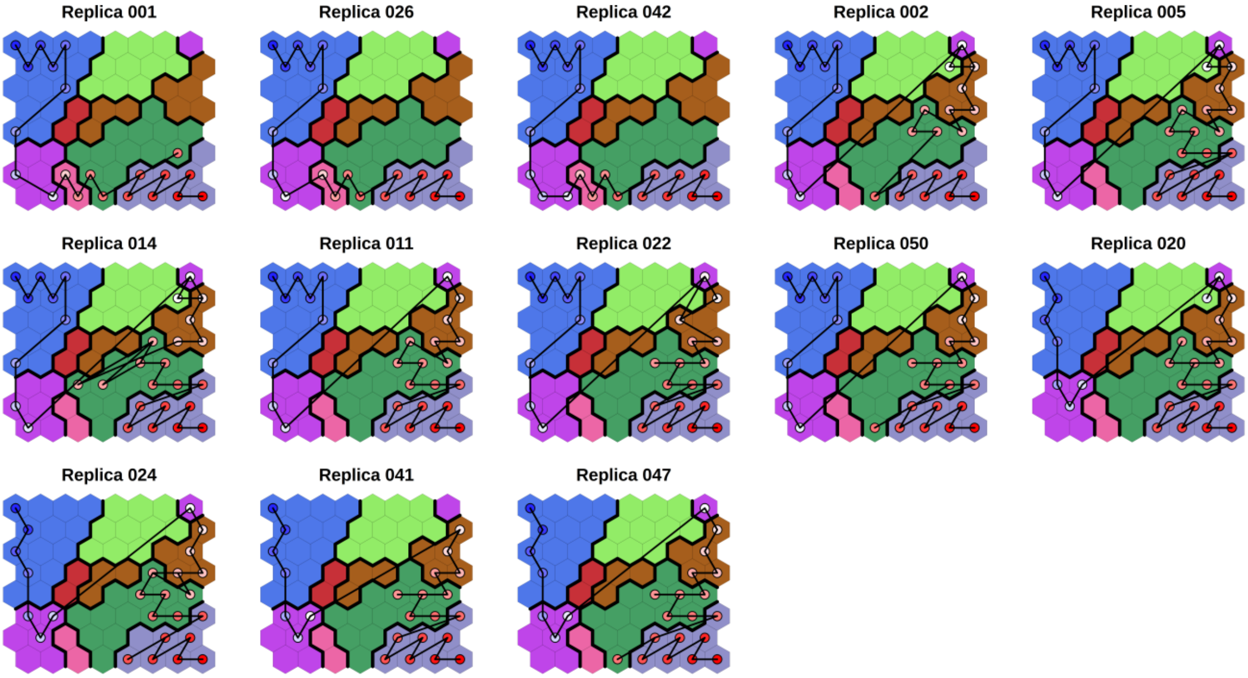

Figure S6: Trace paths on the trained SOM for simulations following pathways 1.

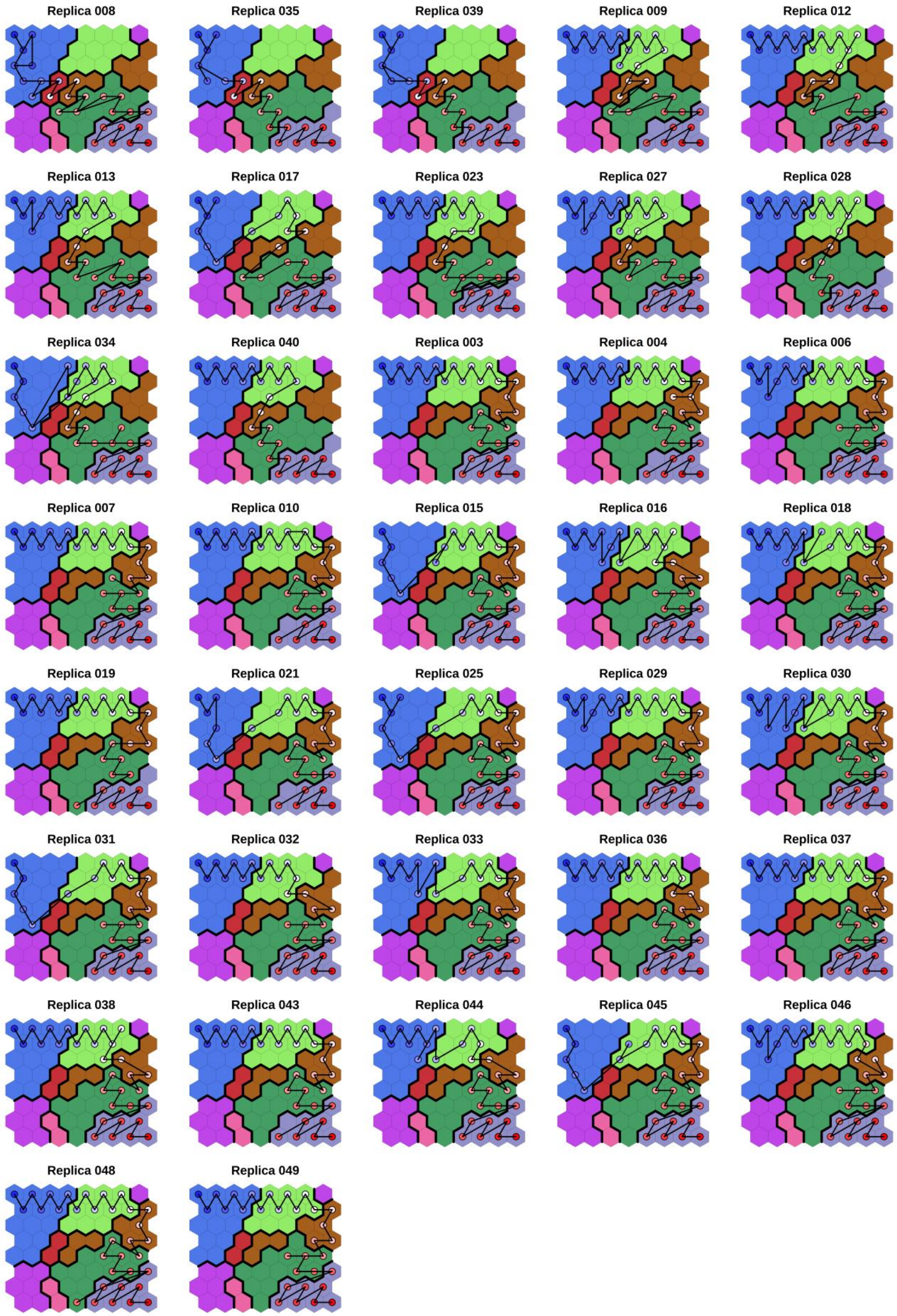

Figure S7: Trace paths on the trained SOM for simulations following pathways 2.

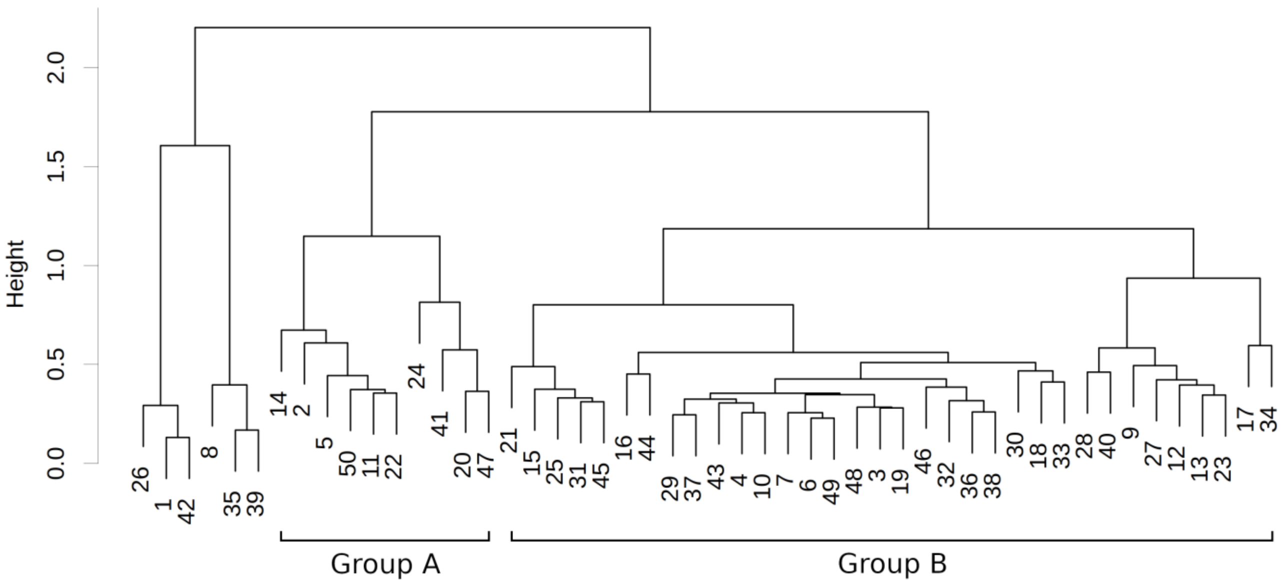

Figure S8: Dendrogram of hierarchical clustering of the pathways followed by different replicas.

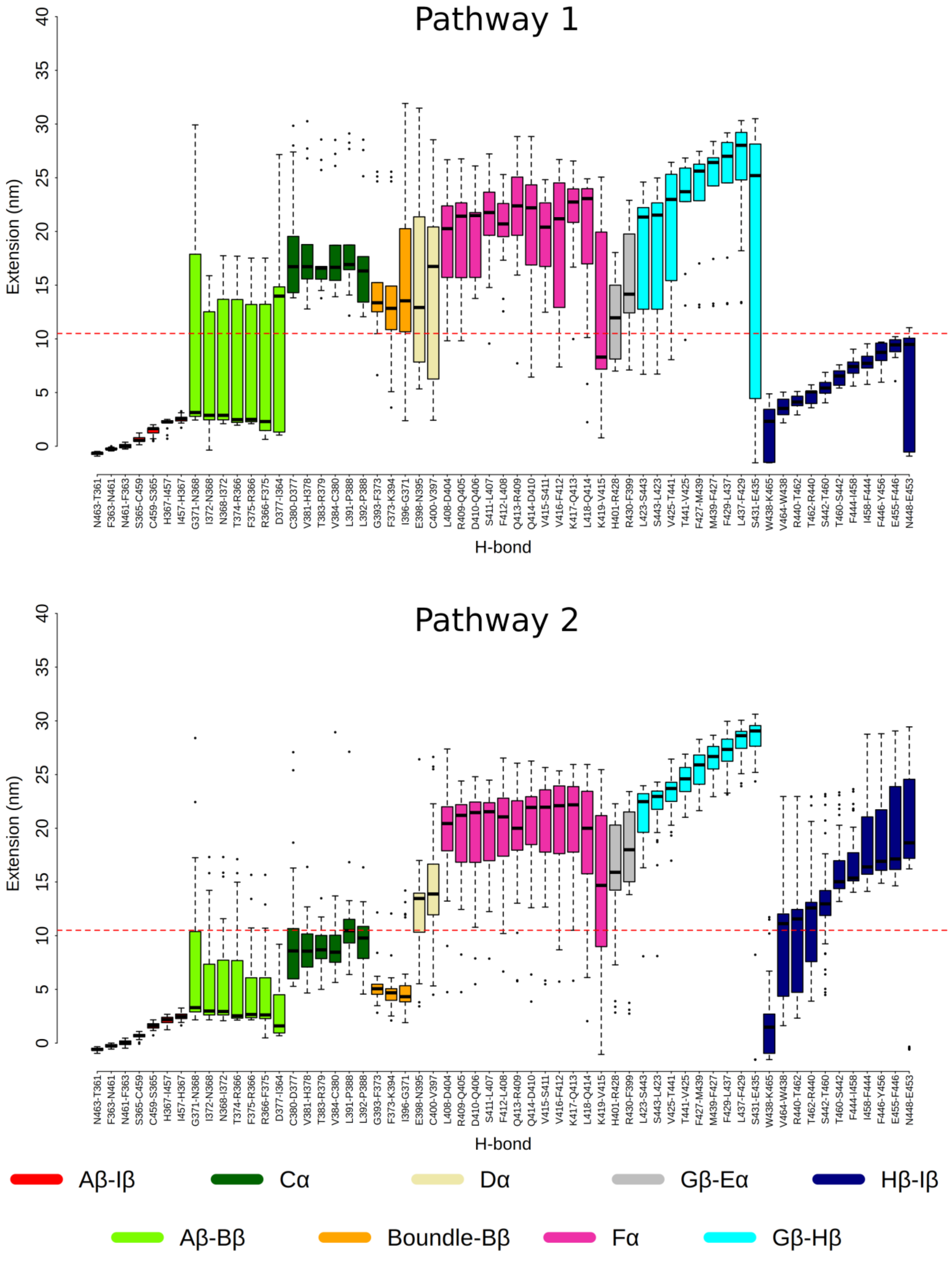

Figure S9: Boxplots reporting the extension of the protein in correspondence of the last frame where each native H-bond was recorded in the different replicas. Simulations following pathway 1 on top, simulations following pathway 2 at bottom. Boxplots are colored according to the secondary structures they belong to.

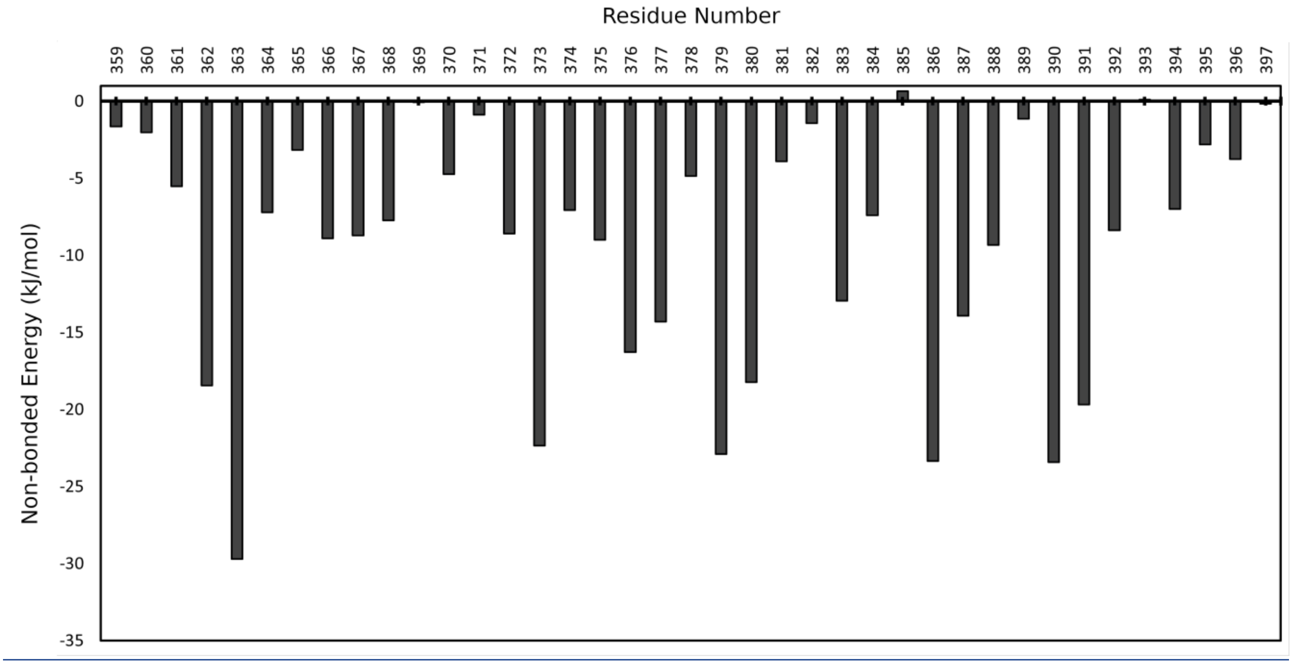

Figure S10: Per-residue non-bonded energy from decomposition analysis. Only residues from the N-terminal lobe are included.

Table S1: Average percentage of secondary structure type in structures from each cluster (left) and average radius of gyration for each cluster (right)

	**Secondary Structure composition (%)**								**Radius of Gyration (nm)**
	**α-Helix**	**β-Sheet**	**Helix 5**	**Helix 3**	**β-Bridge**	**Bend**	**Turn**	**Coil**	
**Cluster A**	17.85	33.21	0.01	5.93	2.48	5.98	15.78	26.75	1.86
**Cluster B**	11.31	24.16	0.05	2.15	0.96	5.62	10.41	53.34	4.48
**Cluster C**	12.52	15.59	0.44	4.63	1.35	8.28	12.36	52.84	3.79
**Cluster D**	4.44	16.44	0.24	1.66	0.33	5.21	6.22	73.45	6.26
**Cluster E**	2.73	34.47	0.27	2.96	0.94	8.66	12.05	45.92	5.11
**Cluster F**	0.37	5.51	0.01	0.52	0.42	4.37	3.48	93.31	8.23
**Cluster G**	8.56	0.18	0.12	4.05	2.05	6.62	9.83	76.59	6.74
**Cluster H**	0.03	0.04	0	0.08	0.03	0.62	0.26	106.95	10.39

REFERENCES

(1) Pandini, A.; Fornili, A.; Fraternali, F.; Kleinjung, J. GSATools: Analysis of Allosteric Communication and Functional Local Motions Using a Structural Alphabet. *Bioinformatics* **2013**, *29* (16), 2053–2055. https://doi.org/10.1093/bioinformatics/btt326.

(2) Kabsch, W.; Sander, C. Dictionary of Protein Secondary Structure: Pattern Recognition of Hydrogen-Bonded and Geometrical Features. *Biopolymers* **1983**, *22* (12), 2577–2637. https://doi.org/https://doi.org/10.1002/bip.360221211.
